# Comparison of 2 modern swept-source optical biometers—IOLMaster 700 and Anterion

**DOI:** 10.1007/s00417-022-05870-9

**Published:** 2022-10-29

**Authors:** Achim Langenbucher, Nóra Szentmáry, Alan Cayless, Jascha Wendelstein, Peter Hoffmann

**Affiliations:** 1 Department of Experimental Ophthalmology, Saarland University, Kirrberger Str 100 Bldg. 22, 66424 Homburg, Saar, Germany; 2 Dr. Rolf M. Schwiete Center for Limbal Stem Cell and Aniridia Research, Saarland University, Homburg, Saar, Germany; 3 Department of Ophthalmology, Semmelweis-University, Mária u. 39, 1085 Budapest, Hungary; 4 School of Physical Sciences, The Open University, Milton Keynes, UK; 5 Department of Ophthalmology, Johannes Kepler University, Linz, Austria; 6 Augen- und Laserklinik Castrop-Rauxel, Castrop-Rauxel, Germany

**Keywords:** Optical biometer, Swept-source OCT, IOLMaster700, Anterion, Power vector analysis, Intraocular lens power calculation

## Abstract

**Purpose:**

To compare biometric measures from 2 modern swept-source OCT biometers (IOLMaster700 (Z, Carl-Zeiss-Meditec) and Anterion (H, Heidelberg Engineering)) and evaluate the effect of measurement differences on the resulting lens power (IOLP).

**Methods:**

Biometric measurements were made on a large study population with both instruments. We compared axial length (AL), central corneal thickness (CCT), anterior chamber depth (ACD), lens thickness (LT) and corneal front and back surface curvature measurements. Corneal curvature was converted to power vectors and total power derived using the Gullstrand formula. A paraxial lens power calculation formula and a prediction for the IOL axial position according to the Castrop formula were used to estimate differences in IOLP targeting for emmetropia.

**Results:**

There were no systematic differences between measurements of AL (− 0.0146 ± 0.0286 mm) and LT (0.0383 ± 0.0595 mm), whereas CCT yielded lower (7.8 ± 6.6 µm) and ACD higher (0.1200 ± 0.0531 mm) values with H. With H, CCT was lower for thicker corneas. The mean corneal front surface radius did not differ (− 0.4 ± 41.6 µm), but the corneal back surface yielded a steeper radius (− 397.0 ± 74.6 µm) with H, giving lower mean total power (− 0.3469 ± 0.2689 dpt). The astigmatic vector components in 0°/90° and 45°/135° were the same between both instruments for the front/back surface or total power.

**Conclusion:**

The biometric measures used in standard formulae (AL, corneal front surface curvature/power) are consistent between instruments. However, modern formulae involving ACD, CCT or corneal back surface curvature may yield differences in IOLP, and therefore, formula constant optimisation customised to the biometer type is required.


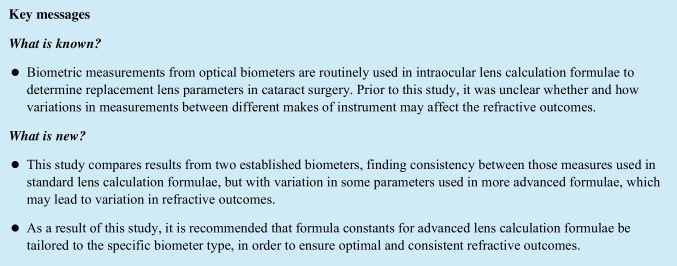


## Introduction

The last decade has seen significant progress in developing new intraocular lens (IOL) concepts and philosophies in cataract surgery, including multifocal (trifocal or quadrifocal) lenses, enhanced depth of focus lenses, monofocal plus lenses or (enhanced) monovision where different lens concepts may be combined (mix and match) to maintain a continuously good imaging performance from far distance to intermediate or even near distance.

These new developments have mostly been enabled by the significant improvements in the quality, repeatability and reliability of the biometric measures as made with modern optical biometers [1, 2]. Additionally, intraocular lens power (IOLP) calculation concepts have also been significantly improved over the same timescale. Many scientific studies investigating the differences in the biometric measures between optical biometers are available in the literature [3–9]. Today, with modern optical biometers based mostly on optical coherence tomography or partial coherence interferometry/reflectometry, all relevant distances in the eye can be measured—including axial length (AL), central corneal thickness (CCT), anterior chamber depth measured from the corneal front apex to the front apex of the crystalline lens (ACD) or alternatively the aqueous depth measured from the corneal endothelium to the lens front apex (AQD), as well as the central thickness of the crystalline lens (LT). Corneal curvature is measured mostly from the anterior surface (flat radius R1a at axis Aa and steep radius R2a), but with some advanced biometers also from the corneal back surface (flat radius R1p at axis Ap and steep radius R2p) [1, 2]. The latest generation of IOLP calculation formulae is adapted to these new features of the biometers and considers—besides axial length and corneal power—additional measures for a more reliable prediction of the IOLP [10]. Therefore, it has been possible over the last decade to significantly reduce the formula prediction error (predError) defined as the deviation of the achieved refractive outcome spherical equivalent (SEQ) from the formula predicted refraction (predSEQ) in a well-selected study population, with up to 50% or 85% resulting in outcomes within limits of ± ¼ dpt or ± ½ dpt [11].

However, we have to be aware that the measures do not match perfectly, even with modern high-end optical biometers, and therefore, the instruments cannot be used interchangeably. It is well known that measures such as AL or CCT give mostly consistent results between instruments [5], whereas other measures such as corneal curvature data, ACD or LT show a larger degree of variation [12]. As a consequence, if a biometer is replaced by another model in a clinical environment, either the biometric measures should be transformed to correspond to the respective measures of the previously used biometer or the formula constants should be adapted to obtain the best results with the replacement device [13].

The IOLMaster 700 (Carl-Zeiss-Meditec, Jena, Germany) which is based on swept-source OCT technology was launched in 2014 to the European market. In addition to the measurement of all distances in the eye (AL_Z_, CCT_Z_, ACD_Z_, AQD_Z_, LT_Z_), a plug-in keratometer is integrated which measures the corneal front surface curvature at 18 locations and reads out the relevant data for IOLP calculation (R1a_Z_, Aa_Z_, R2a_Z_) [2]. In addition, corneal thickness is captured in a central region of about 4.5 mm in diameter with a scanning OCT, and in combination with the keratometric measures, the curvature of the corneal back surface (R1p_Z_, Ap_Z_ and R2p_Z_) is provided. The Anterion (Heidelberg Engineering, Heidelberg, Germany) was launched in 2018 and is solely based on swept-source OCT technology. In addition to all relevant distances in the eye such as AL_H_, CCT_H_, ACD_H_, AQD_H_ and LT_H_, the Anterion acts as an anterior segment tomographer [5], and the topography of the corneal front and back surface is provided at thousands of locations. The respective corneal front and back surface curvature data (R1a_H_, Aa_H_, R2a_H_; R1p_H_, Ap_H_, R2p_H_) used for IOLP calculation are directly extracted from the topography of both corneal surfaces.

The *purpose of the present study* was

- To select paired samples of biometric measurements derived from the IOLMaster and the Anterion swept-source optical biometer in a large cataractous population
- To compare the relevant biometric distances AL, CCT, ACD and LT between both instruments
- To analyse corneal curvature of the front and back surface, to extract the spherocylindric equivalent power of the cornea derived from a thick lens model using the Gullstrand formula and to compare the power vector components
- To predict and compare the IOLP from the biometric measures of the IOLMaster and the Anterion using a modern IOLP calculation formula based on a thick lens concept for the cornea, using formula constants for a modern IOL

## Materials and methods

### Dataset for our analysis

We used a dataset containing in total 981 matched pairs of biometrical measurements from the IOLMaster 700 (Carl-Zeiss-Meditec, Jena, Germany) and the Anterion (Heidelberg Engineering GmbH, Heidelberg, Germany) taken at one clinical centre (Augenklinik Castrop, Castrop-Rauxel, Germany) for this retrospective study. Only one eye per patient was considered, and where measurements of both eyes were available, one eye was selected randomly. All measurements were performed on patients from a cataractous population. Duplicate measurements, pseudophakic eyes or eyes in pharmacologically stimulated mydriasis (pupil width more than 5.2 mm), eyes indexed as being after refractive surgery, or having ectatic corneal diseases (keratoconus, keratoglobus, pellucide marginal degeneration) or other corneal pathologies/ocular trauma were excluded. The data were transferred to.csv data tables using the data export module of the IOLMaster 700/the Anterion software and merged according to the unique patient ID number and eye side (left or right) and the examination date (measurements with both instruments at the same day). Data tables were reduced to the relevant parameters required for our data analysis, consisting of laterality (left or right eye), curvature of the corneal front surface in the flat (R1a) meridian with axis Aa and the steep (R2a) meridian (both in mm), curvature of the corneal back surface in the flat (R1p) meridian with axis Ap and the steep (R2p) meridian (both in mm), AL, CCT, ACD and LT. Parameters measured with the Zeiss IOLMaster were indexed as (.)_Z_ and parameters measured with the Heidelberg-Engineering Anterior were indexed as (.)_H_, respectively. The data were transferred to Matlab (Matlab version 2019b, MathWorks, Natick, USA) for further processing. Incomplete data were eliminated from the dataset. A waiver was provided for this study by the local ethics committee (Ärztekammer des Saarlandes, 157/21).

### Processing of the data

Custom software for data processing and analysis was written in Matlab. For both biometers, we performed the following calculations: mean curvature for the corneal front and back surface was calculated with R12a = 0.5 (R1a + R2a) and R12p = 0.5 (R1p + R2p). Mean keratometric power was derived from the corneal front surface curvature K12 = 0.5 (337.5/R1a + 337.5/R2a). Corneal surface power was expressed in 3 vector components [14, 15] including equivalent power (VEQ) and astigmatic vector considered in the 0/90° meridian (V0) and the oblique 45°/135° meridian (V45) for the front surface:

$$\begin{array}{ccc}VEQa&=&0.5\cdot\left(\frac{\left(nC\;-\;1\right)}{R1a}+\frac{\left(nC\;-\;1\right)}{R2a}\right)\\V0a&=&\left(\frac{\left(nC\;-\;1\right)}{R2a}-\frac{\left(nC\;-\;1\right)}{R1a}\right)\cos\left(2Aa\right)\\V45a&=&\left(\frac{\left(nC\;-\;1\right)}{R2a}-\frac{\left(nC\;-\;1\right)}{R1a}\right)\sin\left(2Aa\right)\end{array}$$

and the back surface:

$$\begin{array}{ccc}VEQp&=&0.5\cdot\left(\frac{\left(nA\;-\;nC\right)}{R1b}+\frac{\left(nA\;-\;nC\right)}{R2p}\right)\\V0p&=&\left(\frac{\left(nA\;-\;nC\right)}{R2b}-\frac{\left(nA\;-\;nC\right)}{R1p}\right)\;\cos\left(2Ap\right)\\V45p&=&\left(\frac{\left(nA\;-\;nC\right)}{R2b}-\frac{\left(nA\;-\;nC\right)}{R1p}\right)\;\sin\left(2Ap\right)\end{array}$$

where *nC* = 1.376 and *nA* = 1.336 denote the refractive index of the cornea and the aqueous humour. The equivalent power of the cornea considered as a thick lens was derived using the classical Gullstrand formula:

$$PEQ=Pa+Pp-Pp\cdot Pa\cdot\frac{CCT}{nC}=\begin{pmatrix}{PEQ}_{1,1}&{PEQ}_{1,2}\\{PEQ}_{2,1}&{PEQ}_{2,2}\end{pmatrix}$$

where the power of the corneal front (*Pa*) and back surface (*Pp*) was replaced by the respective 2 × 2 power matrices [16]:

$$Pa,p=\left(\begin{array}{cc}VEQa,p+V0a,p& V45a,p\\ V45a,p& VEQa,p-V45a,p\end{array}\right)$$

From the 2 × 2 matrix PEQ, the 3 vector components equivalent power (VTPEQ) and the 2 astigmatic components VTP0 and VTP45 were extracted with:

$$\begin{array}{ccc}VTPEQ& =& \left({PEQ}_{\mathrm{1,1}}+{PEQ}_{\mathrm{2,2}}\right)\\ VTP0& =& \left({PEQ}_{\mathrm{1,2}}+{PEQ}_{\mathrm{2,1}}\right)\\ VTP45& =& \left({PEQ}_{\mathrm{1,1}}-{PEQ}_{\mathrm{2,2}}\right)\end{array}$$

The biometric measures from both optical biometers were used to derive the IOLP for emmetropia. For that purpose, we implemented a paraxial formula based on 3 refractive surfaces (corneal front and back surface and IOL as a thin lens). The AL was adjusted (only for lens power calculation) using the Cooke regression formula to correct for long and short eyes. The corneal front and back surface curvature and the CCT were used as measured with the biometers. The effective lens position (axial position of the IOL in the pseudophakic eye with respect to the corneal front apex) was predicted with a linear regression method using AL, ACD and LT and the formula constants as shown for the Castrop formula [13, 14, 17]. Without loss of generality, we performed our IOLP prediction for the formula constant triplets as optimised for the ZCB00 lens (Johnson & Johnson Vision, Santa Ana, USA) extracted from the IOLCon WEB site (https://iolcon.org, accessed on 21.07.2022).

### Statistics and linear modelling of a conversion for power vectors

The overall distribution of data from the entire dataset was described by the mean, standard deviation (SD), median and the lower and upper bounds of the 95% confidence intervals (2.5% quantiles and 97.5% quantile), respectively. Differences in biometric measures between both devices were displayed with Bland-Altmann plots and violin plots, and the distributions of the differences in biometric measures were shown with cumulative density function (CDF) plots. The differences in the power vector components between Anterion and IOLMaster700 are displayed with double-angle plots for the corneal front and back surfaces as well as for the equivalent power derived from the Gullstrand formula.

To gain an impression of how to convert the power vector components of the corneal front and back surfaces and the equivalent power from the IOLMaster700 to the Anterion and vice versa, we developed multiple linear regression models described with transformation matrices. In a first step, to validate the performance of this conversion strategy, we randomly split our dataset into training (70%) and test datasets (30%) for cross-validation. The training data were used to derive the regression model and the test data were used to analyse the performance in terms of the root mean squared prediction error separately for each vector component and using the Euclidian norm (length of the 3-dimensional vector) of the prediction error as a measure for the overall performance. In a second step, the entire dataset was used to provide the matrices for the power vector conversions from IOLMaster700 to Anterion and vice versa.

## Results

After quality approval of the dataset and filtering out incomplete data, a total of *N* = 854 measurements of 403 left and 451 right eyes were enrolled in our study. Table 1 shows the descriptive data of the biometric measures for the entire study population in terms of AL, CCT, ACD, LT and the radii of curvature for the corneal front and back surfaces. The mean keratometric power K12 with the IOLMaster/Anterion were 43.2186 ± 1.5016 dpt (median 43.2138 dpt, 95% confidence interval 40.2193–46.2963 dpt)/44.1668 ± 1.5566 dpt (median 44.1754 dpt, 95% confidence interval 41.2013–47.3452 dpt), respectively.

**Table 1:** Explorative data of ocular biometry in the cataract population. The upper section refers to the biometric measurement with the IOLMaster, the middle section to the corresponding measurement with the Anterion and the lower section to the difference between the measurements with the Anterion and the IOLMaster700. The differences of R1a, R2a, R1p and R2p are not displayed in the table as the respective axes may not match and differences have to be considered with vector components. The table lists the mean, standard deviation (SD), median and the lower and upper bounds of the 95% confidence interval for the axial length (AL), central corneal thickness (CCT), anterior chamber depth (ACD) and thickness of the crystalline lens (LT) together with the flat and steep radii of the corneal front surface (R1a and R2a) and the corneal back surface (R1p and R2p)

*N* = 854	AL in mm	CCT in mm	ACD in mm	LT in mm	R1a in mm	R2a in mm	R12a	R1p in mm	R2p in mm	R12p
IOLMaster700 (Carl-Zeiss-Meditec)										
Mean	23.8503	0.5544	3.1523	4.6477	7.8186	7.6784	7.7336	7.0797	6.7369	6.9085
SD	1.4927	0.0369	0.3957	0.4282	0.2724	0.2731	0.2631	0.2896	0.2890	0.2755
Median	23.6000	0.5540	3.1600	4.6500	7.8100	7.6500	7.7300	7.0700	6.7300	6.9000
2.5% quantile	21.5285	0.4788	2.4300	3.7400	7.2900	7.1156	7.2300	6.5285	6.1770	6.3685
97.5% quantile	27.1890	0.6262	3.9000	5.5215	8.3915	8.2115	8.2900	7.6900	7.3000	7.4700
Anterion (Heidelberg Engineering)										
Mean	23.8358	0.5466	3.2723	4.6869	7.6509	7.8151	7.7332	6.3571	6.6661	6.5115
SD	1.4903	0.0347	0.4000	0.4267	0.2690	0.2682	0.2594	0.2666	0.2630	0.2585
Mmedian	23.5900	0.5460	3.2756	4.6900	7.6400	7.8100	7.7300	6.3500	6.6600	6.5050
2.5% quantile	21.5240	0.4747	2.5276	3.7870	7.1285	7.3085	7.2385	5.8585	6.1685	6.0200
97.5% quantile	27.1790	0.6142	4.0382	5.5445	8.1915	8.3015	8.2715	6.8600	7.2000	7.0100
Difference Anterion − IOLMaster700										
Mean	− 0.0146	− 0.0078	0.1200	0.0383			− 0.0004			− 0.3970
SD	0.0286	0.0066	0.0531	0.0595			0.0416			0.0746
Median	− 0.0100	− 0.0080	0.1140	0.0500			0.0000			− 0.4000
2.5% quantile	− 0.0700	− 0.0019	0.0368	− 0.1300			− 0.0800			− 0.5400
97.5% quantile	0.0400	0.0030	0.2315	0.0900			0.0800			− 0.2500

**Fig. 1:**
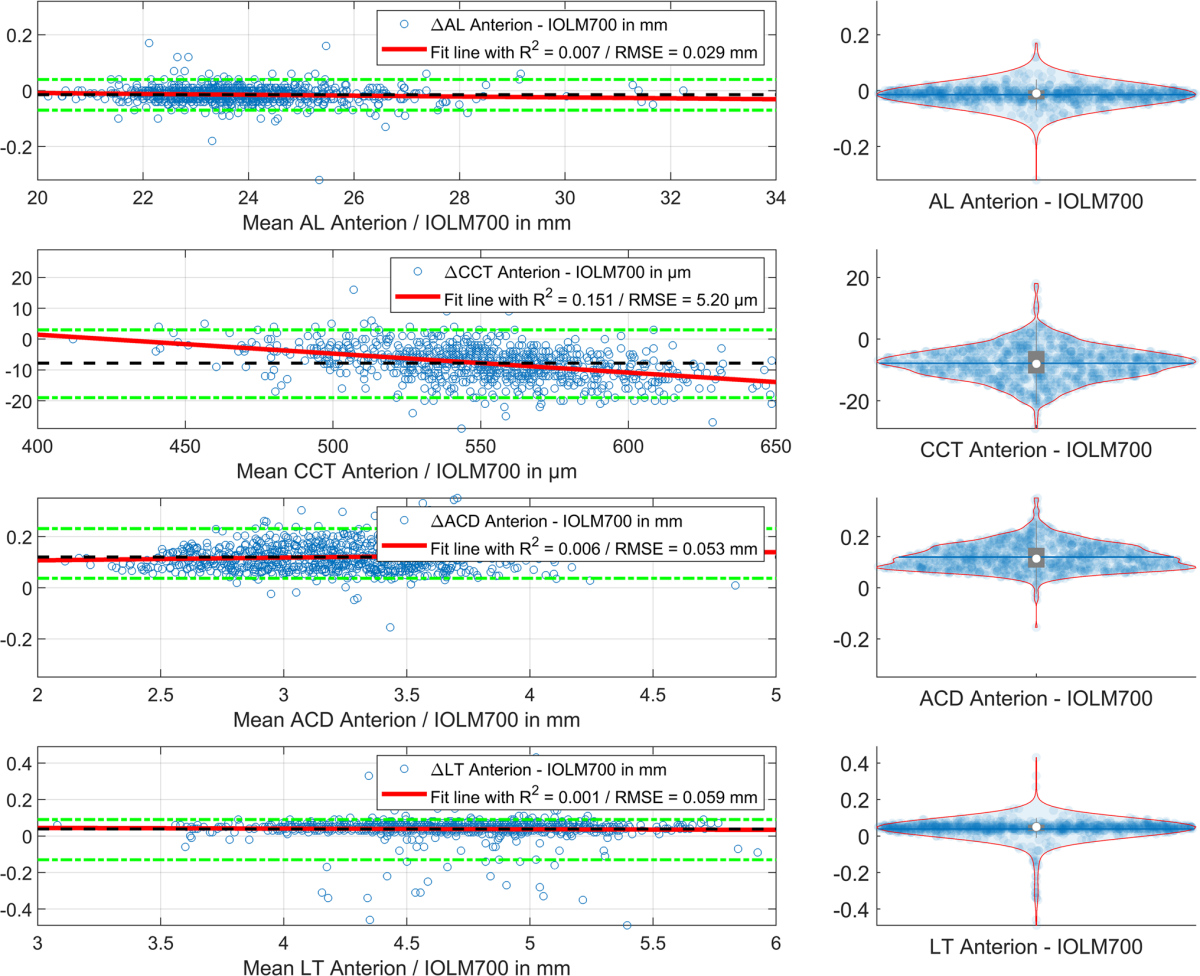
Differences between the biometric distances (axial length (AL), central corneal thickness (CCT), anterior chamber depth (ACD) and crystalline lens thickness (LT)) as measured with the Anterion (Heidelberg Engineering) and the IOLMaster700 (Carl-Zeiss-Meditec). On the left side, the Bland-Altmann plots show the differences between the measurements of both devices versus the mean value including the linear fit lines (solid red lines, with *R*^2^ and root mean squared fit error (RMSE) shown in the legend), the mean value (dashed black lines) and the 95% confidence intervals (dash-dotted green lines, 2.5 and 97.5 quantiles). On the right side, the distributions of the differences are provided with violin plots with mean, median, quartiles and 95% confidence intervals

Figure 1 displays the difference (Anterion − IOLMaster700) over the mean value (Bland-Altmann plot) for the biometric distances in the eye on the left side and the violin plot of the difference on the right side for the AL (upper row of the graph), the CCT (second row), the ACD (third row) and the LT (last row of the graph). It can be seen from the plots that the Anterion yields systematically lower values for CCT and higher values for ACD compared to the IOLMaster700. In addition, CCT measured with the Anterion gives lower values compared to the IOLMaster700 for thicker corneas.

Table 2 lists the components of the power vectors of the corneal front and back surfaces and for the total power derived with the IOLMaster700 and the Anterion, together with the differences between Anterion and IOLMaster700. The mean and median values of the differences are all close to zero except for VEQp and VTPEQ, which indicate that the Anterion yields lower equivalent power values for the corneal back surface and the total corneal power calculated with the Gullstrand formula.

**Table 2:** Descriptive data of the power vector components derived with the IOLMaster700 (upper section), the Anterion (middle section) and the difference in power vector components Anterion − IOLMaster700 (lower section). VEQa/VEQp/VTPEQ refer to the equivalent power for the corneal front surface/corneal back surface/total corneal power, V0a/V0p/VTP0 to the projection of the astigmatism to the 0°/90° meridian for the corneal front surface/corneal back surface/total corneal power and V45a/V45p/VTP45 to the projection of the astigmatism to the 45°/135° meridian for the corneal front surface/corneal back surface/total corneal power respectively. The mean and median values of the differences between both devices are all close to 0, except VEQp and VTPEQ, which show lower values for the Anterion (in bold)

All data in dpt; *N* = 854	VEQa	V0a	V45a	VEQp	V0p	V45p	VTPEQ	VTP0	VTP45
IOLMaster700 (Carl-Zeiss-Meditec)									
Mean	48.6861	0.2863	− 0.0342	− 6.8039	− 0.2545	− 0.0452	42.9962	0.0375	− 0.0788
SD	1.6551	1.2105	0.6863	0.2323	0.1630	0.1140	1.4604	1.0943	0.6215
Median	48.6608	0.2005	− 0.0619	− 0.8057	− 0.2448	− 0.0422	42.9538	− 0.0467	− 0.1034
2.5% quantile	45.3823	− 1.7984	− 1.2611	− 6.2856	− 0.6072	− 0.2839	40.0405	− 1.8313	− 1.2266
97.5% quantile	52.0275	3.0378	1.4493	− 5.3574	0.0369	0.1686	40.9859	2.5937	1.2403
Anterion (Heidelberg Engineering)									
Mean	48.6867	0.3781	0.0160	− 6.1566	− 0.2704	− 0.0044	42.6493	0.1138	0.0118
SD	1.6336	1.1632	0.6500	0.2414	0.1582	0.1013	1.4414	1.0398	0.5831
Median	48.6752	0.2976	0.0000	− 6.1553	− 0.2616	0.0000	42.6071	0.0403	− 0.0116
2.5% quantile	45.4700	− 1.6141	− 1.2057	− 6.6420	− 0.6078	− 0.2042	39.8077	− 1.7274	− 1.0912
97.5% quantile	51.9821	3.2507	1.2989	− 6.7076	0.0102	0.1768	45.5726	2.6765	1.2535
Difference Anterion − IOLMaster700									
mean	0.0006	0.0918	0.0502	** − 0.3527**	− 0.0159	0.0508	** − 0.3469**	0.0763	0.0903
SD	0.2623	0.3264	0.2641	0.0648	0.0686	0.0719	0.2689	0.3127	0.2541
median	0.0000	0.1076	0.0688	** − 0.3502**	− 0.0168	0.0368	** − 0.3470**	0.0915	0.1024
2.5% quantile	− 0.5316	− 0.6334	− 0.5152	− 0.4767	− 0.1384	− 0.1021	− 0.8391	− 0.5919	− 0.4473
97.5% quantile	0.5084	0.7377	0.5712	− 0.2303	0.1240	0.1847	0.1309	0.6983	0.5799

**Fig. 2:**
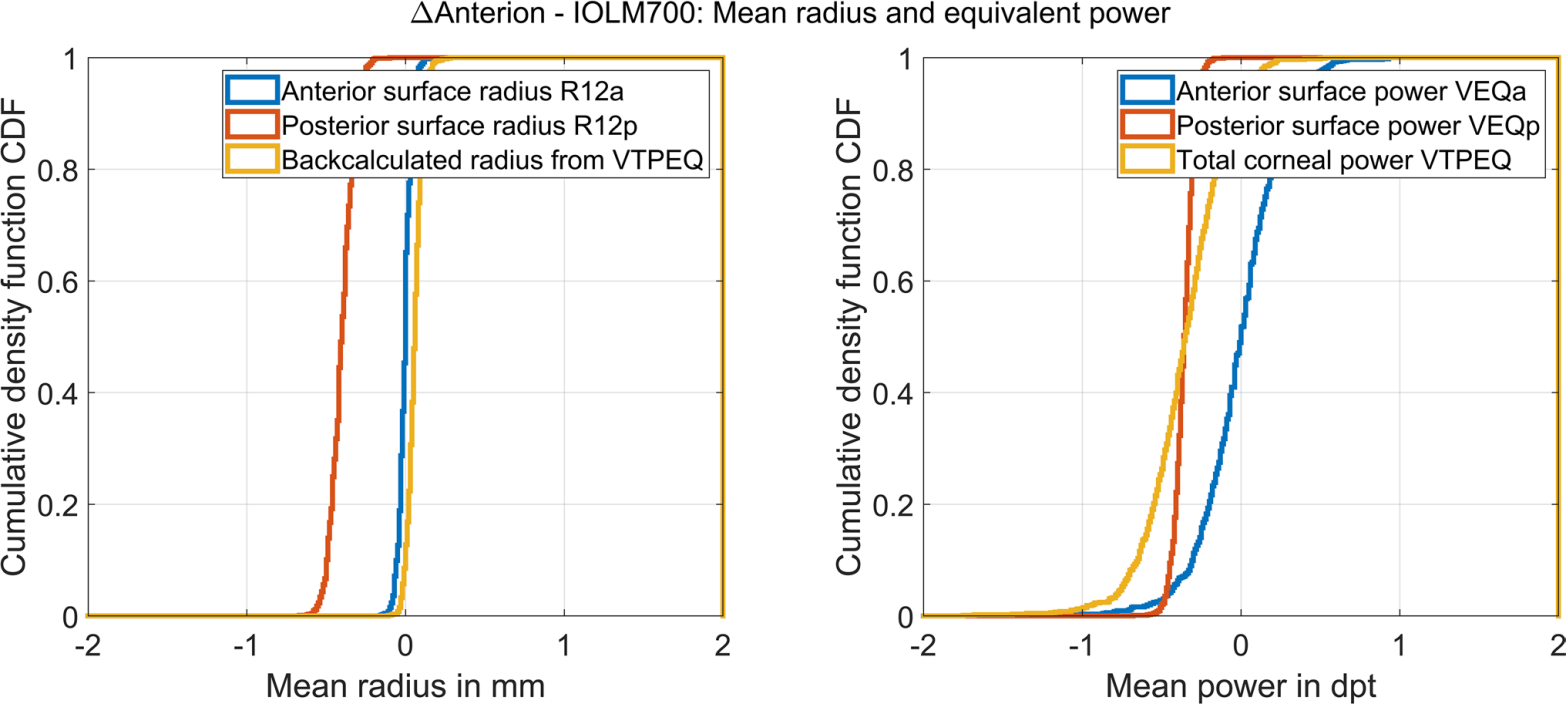
Cumulative density function (CDF) plot for the distributions of the differences (Anterion − IOLMaster700) for R12a, R12p and the radius back-calculated from VTPEQ representing the cornea as a thick lens on the left side and for the mean power VEQa, VEQp and VTPEQ representing the cornea as a thick lens on the right side. From the graph, we see that R12a and VEQa of the corneal front surface are quite similar with both instruments, but the R12p/VEQp of the corneal back surface yields lower/more negative values compared with the IOLMaster700 biometer. As a consequence, the total corneal power VTPEQ calculated from the Gullstrand formula (yellow line on the right plot) gives systematically lower values for the Anterion compared to the IOLMaster700

In Fig. 2, the cumulative density function (CDF) plot for the distributions of the differences (Anterion − IOLMaster700) are shown for R12a, R12p and the radius back-calculated from VTPEQ (representing the cornea as a thick lens) on the left graph and for the equivalent power components of the power vector VEQa, VEQp and VTPEQ (representing the cornea as a thick lens) on the right graph. From the graph, we see that R12a and VEQa of the corneal front surface are quite similar with both instruments, but the R12p/VEQp of the corneal back surface yields lower/more negative values compared to the IOLMaster700 biometer. As a consequence, the total corneal power VTPEQ calculated from the Gullstrand formula (yellow line on the right plot) gives systematically lower values for the Anterion compared to the IOLMaster700.

**Fig. 3:**
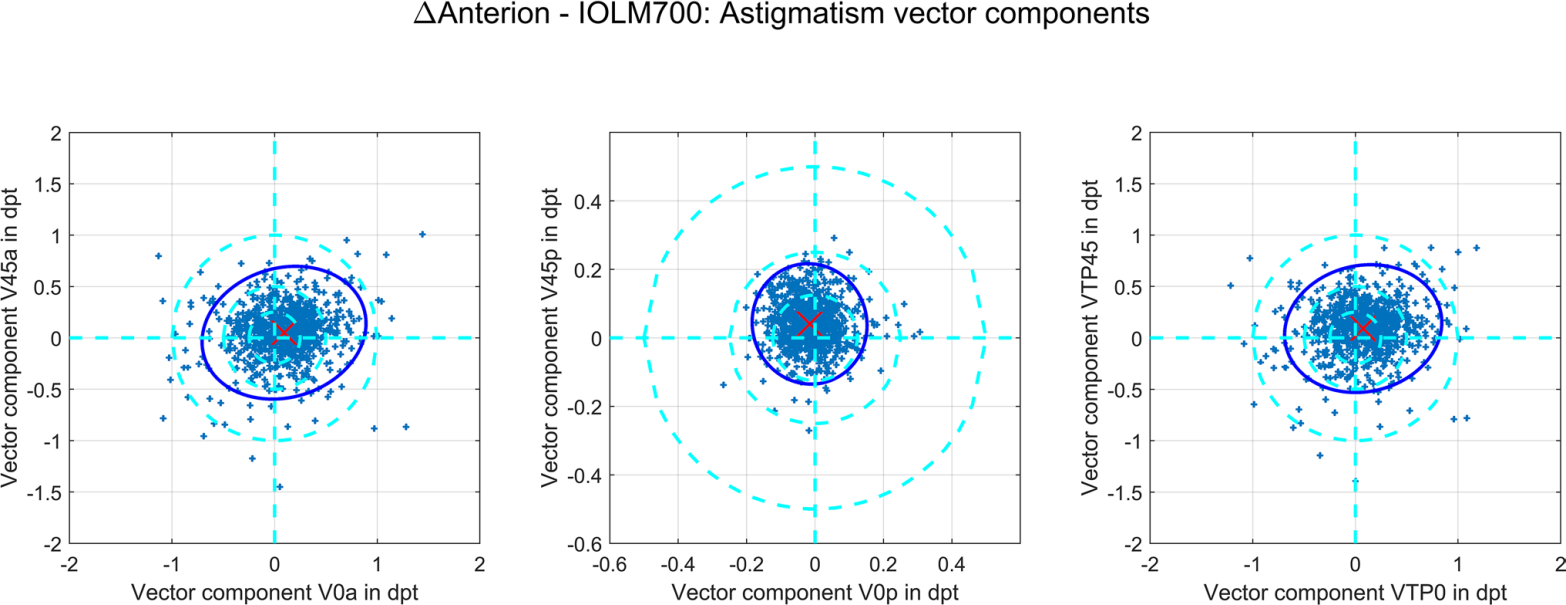
The double-angle plot for the difference (Anterion − IOLMaster700) of the astigmatic components of the power vector. The vector components for the corneal front and back surface are plotted on the left and middle graphs, and on the right graph, the astigmatic components of the power vector are shown for the total power derived from the Gullstrand formula considering the cornea as a thick lens. The error ellipses shown in the plot (blue ellipses) are derived from the covariance matrices, and the centroids (red x) indicate the mean differences of the vector components in 0°/90° (horizontal direction) and in 45°/135° (vertical direction). From the graphs, we can see that the Anterion measures a slightly larger cylinder against the rule for the corneal back surface (centroid in the middle plot shifted to above) which also affects a slightly larger cylinder against the rule for the total corneal power (centroid in the right plot shifted to above)

Figure 3 displays the double-angle plot for the difference (Anterion − IOLMaster700) of the astigmatic components of the power vector. In the left and middle graphs, the vector components for the corneal front and back surfaces are plotted, and on the right graph, the astigmatic components of the power vector are shown for the total power derived from the Gullstrand formula considering the cornea as a thick lens. The error ellipses shown in the plot (blue ellipses) are derived from the covariance matrices, and the centroids (red x) indicate the mean differences of the vector components in 0°/90° (horizontal direction) and in 45°/135° (vertical direction). It can be seen from the graphs that the Anterion measures a slightly larger cylinder against the rule for the corneal back surface (centroid (x/y: − 0.0159/0.0408 dpt) in the middle plot shifted to above), which also affects a slightly larger cylinder against the rule for the total corneal power (centroid in the right plot shifted to above). The respective centroids for the corneal front surface astigmatism (x/y: 0.0918/0.0502 dpt) and for the total power (x/y: 0.0763/0.0903 dpt) are more centred with respect to the data scatter.

The linear model for converting the components of the power vector for the corneal front surface from IOLMaster700 to Anterion and vice versa reads:

$$\begin{array}{ccc}\begin{pmatrix}{VEQa}_H\\{V0a}_H\\{V45a}_H\end{pmatrix}&=&\begin{pmatrix}1.2298\\0.6591\\0.3079\end{pmatrix}+\begin{pmatrix}0.9748&-0.0060&0.0075\\-0.0111&0.9107&0.0075\\-0.0054&0.0053&0.8745\end{pmatrix}\cdot\begin{pmatrix}{VEQa}_Z\\{V0a}_Z\\{V45a}_Z\end{pmatrix}\\\begin{pmatrix}{VEQa}_Z\\{V0a}_Z\\{V45a}_Z\end{pmatrix}&=&\begin{pmatrix}-1.2298\\-0.6591\\-0.3079\end{pmatrix}+\begin{pmatrix}1.0259&0.0068&-0.0089\\0.0125&1.0982&-0.0095\\0.0063&-0.0066&1.1435\end{pmatrix}\cdot\begin{pmatrix}{VEQa}_H\\{V0a}_H\\{V45a}_H\end{pmatrix}\end{array}$$

with a loglikelihood objective function value after the final iteration of − 2659.

The respective linear model for converting the components of the power vector for the corneal back surface from IOLMaster700 to Anterion and vice versa reads:

$$\begin{array}{ccc}\begin{pmatrix}{VEQp}_H\\{V0p}_H\\{V45p}_H\end{pmatrix}&=&\begin{pmatrix}-0.3482\\-0.0496\\0.1572\end{pmatrix}+\begin{pmatrix}1.0000&0.0078&0.0562\\-0.0010&0.8805&0.0573\\0.0223&0.0038&0.6958\end{pmatrix}\cdot\begin{pmatrix}{VEQp}_Z\\{V0p}_Z\\{V45p}_Z\end{pmatrix}\\\begin{pmatrix}{VEQp}_Z\\{V0p}_Z\\{V45p}_Z\end{pmatrix}&=&\begin{pmatrix}0.3482\\0.0496\\-0.1572\end{pmatrix}+\begin{pmatrix}1.0018&-0.0086&-0.0802\\0.0032&1.1362&-0.0938\\-0.0321&-0.0059&1.4403\end{pmatrix}\cdot\begin{pmatrix}{VEQp}_H\\{V0p}_H\\{V45p}_H\end{pmatrix}\end{array}$$

with a loglikelihood objective function value after the final iteration of 3425.2.

The respective linear model for converting the components of the power vector for the total corneal power (using the Gullstrand formula) from IOLMaster700 to Anterion and vice versa reads:

$$\begin{array}{ccc}\begin{pmatrix}{VTPEQ}_H\\{VTP0}_H\\{VTP45}_H\end{pmatrix}&=&\begin{pmatrix}0.8804\\0.5128\\0.4241\end{pmatrix}+\begin{pmatrix}0.9715&-0.0032&0.0058\\-0.0100&0.9114&0.0154\\-0.0080&0.0051&0.8570\end{pmatrix}\cdot\begin{pmatrix}{VTPEQ}_Z\\{VTP0}_Z\\{VTP45}_Z\end{pmatrix}\\\begin{pmatrix}{VETPQ}_Z\\{VTP0}_Z\\{VTP45}_Z\end{pmatrix}&=&\begin{pmatrix}-0.8804\\-0.5128\\-0.4241\end{pmatrix}+\begin{pmatrix}1.0293&0.0037&-0.0070\\0.0112&1.0974&-0.0198\\0.0096&-0.0064&1.1669\end{pmatrix}\cdot\begin{pmatrix}{VTPEQ}_H\\{VTP0}_H\\{VTP45}_H\end{pmatrix}\end{array}$$

with a loglikelihood objective function value after the final iteration of − 192.6.

In the next step, the data were split into training (70%) and test (30%) datasets (600 records in the training dataset and 254 in the test dataset) for cross-validation. The models to transform the power vectors for the corneal front and back surfaces and the total power from the measurements of the IOLMaster700 to the Anterion were fitted using the training data and later evaluated using the test data. The Euclidian norm of the fit error (power vector measured with the Anterion minus the power vector prediction from the respective components of the IOLMaster using the linear regression model) yielded 0.4090 ± 0.2211 dpt (median 0.3702 dpt, 95% confidence interval 0.1112–0.8936 dpt) for the anterior surface of the cornea, 0.0986 ± 0.0460 dpt (median 0.0929 dpt, 95% confidence interval 0.0263–0.2014 dpt) for the posterior surface of the cornea and 0.3999 ± 0.2076 dpt (median 0.3505 dpt, 95% confidence interval 0.1242–0.8719 dpt) for the total power. When applied to the training dataset, the Euclidian norm of the fit error yielded 0.4093 ± 0.2431 dpt (median 0.3538 dpt, 95% confidence interval 0.1037–1.0547 dpt) for the anterior surface of the cornea, 0.0986 ± 0.0460 dpt (median 0.0929 dpt, 95% confidence interval 0.0263–0.2014 dpt) for the posterior surface of the cornea and 0.3999 ± 0.2076 dpt (median 0.3505 dpt, 95% confidence interval 0.1242–0.8719 dpt) for the total power. This means that the uncertainty (standard deviation) of the power vector prediction for the test dataset is about 0.21–0.22 dpt for the corneal front surface and the total power and about 0.05 dpt for the corneal back surface both for the training and for the test dataset, proving that there is no noticeable overfitting.

**Fig. 4:**
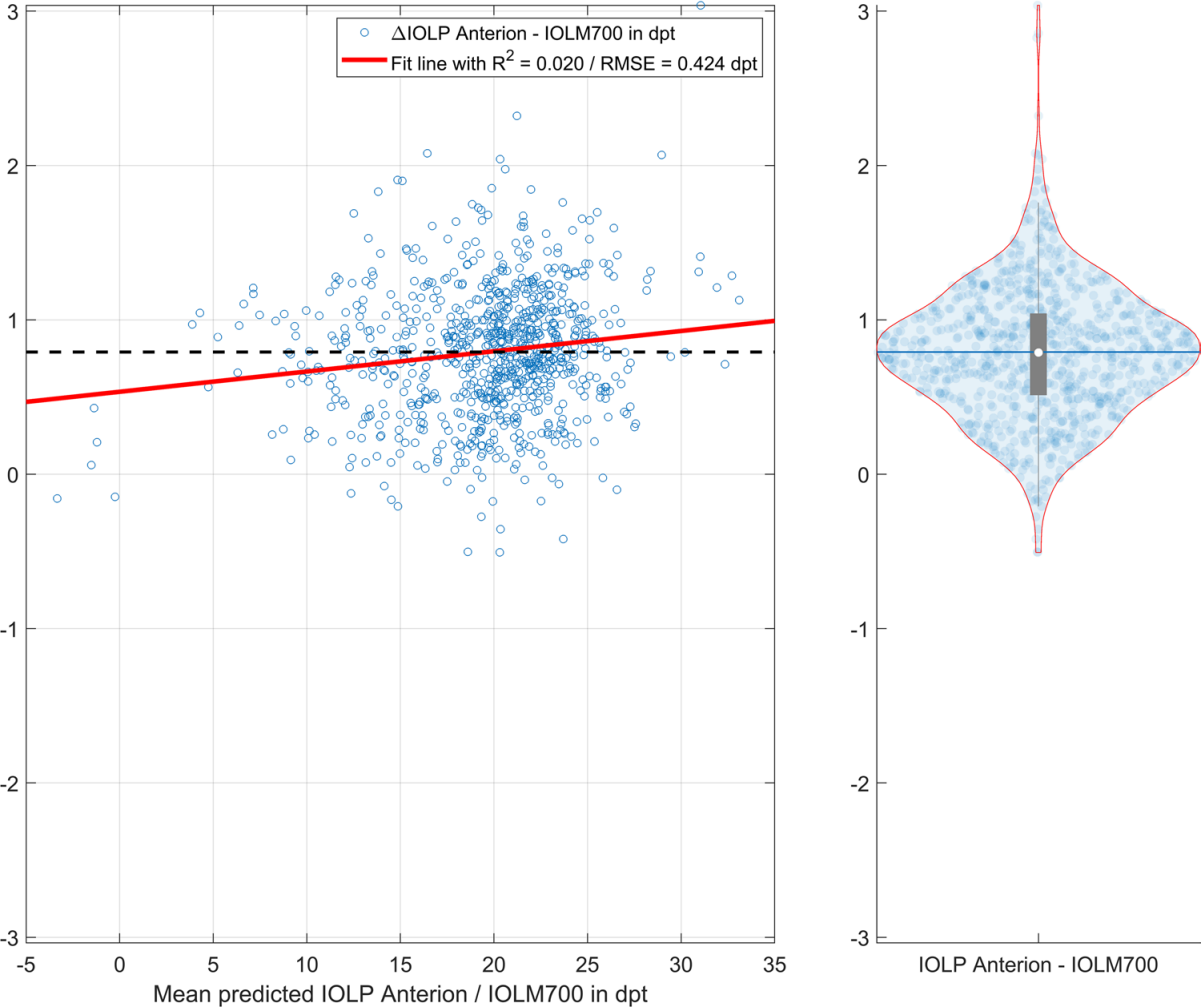
Differences between the predicted intraocular lens power IOLP derived from the biometric measures of the Anterion and the IOLMaster700 for emmetropia using a paraxial lens power calculation formula with 3 refractive surfaces (corneal front and back surface and the intraocular lens as a thin lens). For the IOLP prediction, the axial length AL, central corneal thickness CCT, anterior chamber depth ACD and lens thickness LT are used together with the mean corneal curvature of the corneal front surface R12a and back surface R12p. The axial position of the lens was predicted with a regression setup as described for the Castrop formula. The Bland-Altmann plot on the left graph shows the difference in IOLP versus the mean IOLP together with a best fit regression line (solid red line, *R*^2^ and root mean squared fit error RMSE in the legend) and the mean value (black dashed line), and the right graph displays the violin plot for the distribution of the difference in IOLP with mean, median, quartiles and 95% confidence intervals

In the final step, the IOLP for a plano target refraction was calculated based on the biometric measures from the Anterion and IOLMaster700 devices. Using a paraxial lens power calculation formula with 3 refractive surfaces (corneal front and back surfaces and the intraocular lens as a thin lens) and a prediction scheme for the axial position of the IOL according to the Castrop formula, the IOLP was determined from AL, CCT, ACD and LT together with the mean corneal curvatures R12a and R12p. The IOLP calculation yielded 20.0095 ± 4.6577 dpt (median 20.7885 dpt, 95% confidence interval 9.3964 to 27.2703 dpt) for the Anterion and 19.2180 ± 4.5971 dpt (median 19.9414 dpt, 95% confidence interval 8.7290 to 26.6026 dpt) for the IOLMaster700. The difference in IOLP comparing the Anterion and the IOLMaster700 (Anterion − IOLMaster700) was 0.7915 ± 0.4281 dpt (median 0.7885 dpt, 95% confidence interval 0.0184 to 1.6576 dpt). Figure 4 shows the Bland-Altmann plot on the left graph with the difference in IOLP versus the mean IOLP together with a best fit regression line (solid red line, *R*^2^ and root mean squared fit error RMSE in the legend) and the mean value (black dashed line). On the right side of Fig. 4, the violin plot displays the distribution of the difference in IOLP with mean, median, quartiles and 95% confidence intervals. We can directly see from the graph that—using identical formula constants for both biometers—the IOLP values derived with the Anterion biometric measures are on average systematically higher compared to the respective values derived with the IOLM700 measures.

## Discussion

Ocular biometry as the fundamental basis for IOL power calculation is one of the most essential mosaic pieces in modern cataract surgery. Previously, with ultrasound biometers, refractive outcome variations of 1 or 2 dpt from the target refraction were quite usual. With modern biometry and advanced lens power calculation concepts, root mean squared prediction errors have been reduced to 0.3–0.4 dpt, and the demands of the patients and the surgeons increased significantly [10, 18]. The newest generation of optical biometers is not restricted to measurements of AL, ACD and corneal front surface, but also provides data on CCT and LT, and some of them even provide data on the corneal back surface curvature [2].

In the present paper, we set out to compare the biometric measures of 2 modern optical biometers both based on a swept-source OCT technology. Both instruments measure the curvature of both corneal surfaces in addition to AL, CCT, ACD and LT. The IOLMaster 700 uses a plug-in keratometer which reads corneal front surface curvature at 6 locations on each of 3 concentric rings (18 points in total). With the swept-source OCT, the corneal thickness is derived in a central region of about 4 to 4.5 mm, and both measurement modalities are registered to provide the thick lens model of the cornea. In contrast, the focus of the Anterion is mostly as an anterior segment analyser with a full-size measurement of the cornea and anterior eye segment, and an axial length measurement is included to make the Anterion an optical biometer suitable for preoperative cataract biometry. In contrast to the IOLMaster, no classical keratometer is implemented and the corneal front surface curvature is extracted from the OCT data.

In a large cataractous population, measurements were performed with both biometers on the same day. The measurement results were extracted using the download function of the respective software and merged. As the most relevant data for lens power calculation, the AL, CCT, ACD and LT plus the corneal front and back surface curvature were compared. As the locations of the flat and steep meridians for the corneal front and back surfaces do not necessarily coincide, the radii of curvature cannot be directly compared [12]. Instead, we performed a vector analysis and decomposed the corneal front and back surface power into 3D power vectors [15] including equivalent power and projections of the astigmatism to the 0°/90° and to the 45°/135° meridians. To properly compare the total corneal power, we implemented the classical Gullstrand formula and generalised it to spherocylindrical surfaces using 2 × 2 power matrices [16]. Using this method, we could easily access the respective 3D power vector of the equivalent power (independent of any internal calculation scheme implemented in the biometer software), which is referenced to the secondary principal plane of the cornea. This equivalent power derived from the Gullstrand formula is based on the curvature of both corneal surfaces, the CCT and the refractive index of the cornea and aqueous humour, but is independent from any keratometer index [12].

We discovered that on average, the AL and LT measures match pretty well without any systematic offset, but the CCT and ACD measures show systematic differences between both instruments (Fig. 1). Interestingly, the extreme values or outliers in the Bland-Altmann plot for LT are mostly in the negative range, which could be the result of inaccuracies in the surface detection process for the lens front and/or back surface. CCT in general shows lower values with the Anterion compared to the IOLMaster700, and the regression line in the graph indicates that the difference between both instruments increases for thicker corneas. However, this difference in CCT does noticeably affect the resulting IOLP in lens power calculation. In contrast, the ACD value derived with the Anterion seems to be systematically larger compared to the IOLMaster700 and this is clinically relevant for lens power calculation concepts which consider phakic ACD, but from the regression line, we can see that there is no trend error for larger or smaller values of ACD. Surprisingly, from the mean radius of curvature shown in Fig. 2, we can see that there is no systematic difference between both instruments in the readings of corneal front surface curvature, but the Anterion yields systematically lower values for the corneal back surface radius of curvature [19], resulting in a more negative mean power of the corneal back surface. This difference could be caused by differences in the implementation of inverse raytracing for compensation of image distortion when deriving the geometric corneal back surface profile from the OCT images. The mean difference for the front surface equivalent component of the power vector VEQa is quite close to 0 (compare Table 2) whereas the respective value for the back surface power VEQp is systematically negative. As a consequence, the respective difference for the total power derived from the Gullstrand formula (VTPEQ) also yields systematically negative values. Analysing the 2 astigmatic components of the 3D power vector for the corneal front and back surface and comparing the results of both devices under test, we can see in the double-angle graph in Fig. 3 that there are only very slight shifts of the centroids from the centre. This means that there is no systematic difference between the Anterion and the IOLMaster700 for the astigmatism of the corneal front or back surface and total corneal power.

A simple conversion for the 3D power vector from Anterion to IOLMaster700 or vice versa is derived using a multilinear regression setup for the corneal front and back surface as well as for the total corneal power based on the Gullstrand formula. The values on the diagonals of the 3 × 3 matrices are all close to 1, indicating that we are mostly dealing with a 1:1 conversion. The prediction error derived from the cross-validation step is quite moderate and proves that there is no noticeable overfitting of our linear approach. If we simplify these regression models and suppress a cross-talk of the 3 power vector components, the respective linear models read:

$$\begin{array}{ccc}\begin{pmatrix}{VEQa}_H\\{V0a}_H\\{V45a}_H\end{pmatrix}&=&\begin{pmatrix}{1.2398+0.9745\cdot VEQa}_Z\\{0.1176+0.9096\cdot V0a}_Z\\{0.0459+0.8744\cdot V45a}_Z\end{pmatrix}\\\begin{pmatrix}{VEQp}_H\\{V0p}_H\\{V45p}_H\end{pmatrix}&=&\begin{pmatrix}{-0.3484+1.0007\cdot VEQp}_Z\\-0.0459+0.8920\cdot{V0p}_Z\\0.0271+0.6957\cdot{V45p}_Z\end{pmatrix}\\\begin{pmatrix}{VTPEQ}_H\\{VTP0}_H\\{VTP45}_H\end{pmatrix}&=&\begin{pmatrix}{0.8844+0.9714\cdot VTPEQ}_Z\\0.0797+0.9106\cdot{VTP0}_Z\\0.0790+0.8565\cdot{VTP45}_Z\end{pmatrix}\end{array}$$

From that simplified model, we find that the oblique vector components (in 45°/135°) especially for the corneal back surface power vector have a lower weight in the Anterion compared to the IOLMaster700 (0.6957), whereas the 3 weights for the equivalent power components are all in a range from 0.9714 to 1.0007. The respective weights for the power vector components in 0°/90° range between 0.8920 and 0.9106.

Ultimately, the differences in biometric measures comparing the 2 biometers are not relevant to the outcome of the patient. Therefore, we used a general paraxial lens power calculation concept to evaluate the effect of differences in the biometric measures on the resulting lens power. As an example, and without loss of generality, we selected a lens power calculation concept which considers the cornea as a thick lens with its front and back surface curvature and CCT. For the prediction of the axial lens position, we used a concept which considers AL, ACD and LT [13, 14, 18]. For a better comparison, we used identical formula constants (derived from https://IOLCon.org for the ZCB00 Tecnis lens (Johnson & Johnson Vision)) and calculated the lens power for postoperative emmetropia. The IOLP using the Anterion biometric measures is systematically higher compared to the IOLP based on the IOLMaster700 measures. This result is not surprising as the Anterion provides lower values for the corneal back surface curvature and these results in a lower total corneal power. In addition, the mean ACD measured with the Anterion is about 0.1 mm larger which additionally results in larger IOLP values. Overall, this means that the two instruments cannot be used interchangeably [17], and because of the lower total corneal power based on the steeper corneal back surface curvature and the larger ACD, at least for formulae which consider the phakic ACD and/or the corneal back surface shape, the formula constants must be customised for the Anterion to avoid systematic refraction errors after cataract surgery.

In conclusion, optical biometers today yield reliable data on biometric measures used for intraocular lens power calculation. Our study shows that the biometric measures of the Anterion and the IOLMaster700 cannot be used interchangeably. Whereas the results for axial length, corneal front surface curvature and lens thickness are comparable using both devices, the phakic anterior chamber depth is measured deeper and the corneal back surface curvature is steeper/back surface power (and consequently the total power) is more negative with the Anterion, both of which result in a larger intraocular lens power if these parameters are considered by the lens power calculation scheme and the formula constants are not customised. As a consequence, if lens power calculation concepts are used which consider the posterior corneal surface curvature and/or the phakic anterior chamber depth the formula constants should be updated and customised to the specific biometer used, in order to ensure a proper lens power prediction.
